# Effect of Waste Ceramic Powder on Properties of Alkali-Activated Blast Furnace Slag Paste and Mortar

**DOI:** 10.3390/polym13162817

**Published:** 2021-08-22

**Authors:** Gui-Yu Zhang, Yong-Han Ahn, Run-Sheng Lin, Xiao-Yong Wang

**Affiliations:** 1 Department of Architectural Engineering, College of Engineering, Kangwon National University, Chuncheon-si 24341, Korea; zhangguiyu@kangwon.ac.kr (G.-Y.Z.); linrunsheng@kangwon.ac.kr (R.-S.L.); 2 Department of Architectural Engineering, Hanyang University, Ansan-si 15588, Korea; yhahn@hanyang.ac.kr; 3 Department of Integrated Energy and Infra System, College of Engineering, Kangwon National University, Chuncheon-si 24341, Korea

**Keywords:** waste ceramic powder (WCP), alkali-activated slag (AAS), compressive strength, sulfuric acid attack, chloride ion diffusion coefficient

## Abstract

Every year, ceramic tile factories and the iron smelting industry produce huge amounts of waste ceramic tiles and blast furnace slag (BFS), respectively. In the field of construction materials, this waste can be used as a raw material for binders, thus reducing landfill waste and mitigating environmental pollution. The purpose of this study was to mix waste ceramic powder (WCP) into BFS paste and mortar activated by sodium silicate and sodium hydroxide to study its effect on performance. BFS was partially replaced by WCP at the rate of 10–30% by weight. Some experimental studies were conducted on, for example, the fluidity, heat of hydration, compressive strength testing, ultrasonic pulse velocity (UPV), Fourier transform infrared spectroscopy (FTIR), X-ray diffraction (XRD), electrical resistivity, sulfuric acid attack, and chloride ion diffusion coefficient. Based on the results of these experiments, the conclusions are: (1) increasing the amount of waste ceramic powder in the mixture can improve the fluidity of the alkali-activated paste; (2) adding waste ceramic powder to the alkali-activated mortar can improve the resistance of the mortar to sulfuric acid; (3) adding waste ceramic powder to the alkali-activated mortar can increase the diffusion coefficient of chloride ions; (4) the early strength of alkali-activated mortar is affected by the Ca/Si ratio, while the later strength is affected by the change in the Si/Al ratio.

## 1. Introduction

Ordinary Portland cement (OPC) is the most widely used building material in the world. However, the carbon dioxide (CO_2_) emitted from OPC production accounts for approximately 6% of global CO_2_ emissions [1], negatively impacting the global response to climate change. Therefore, the governments and industries in various countries have been encouraging the cement industry to reduce its CO_2_ emissions. There are two ways to reduce cement usage: the first is to use mixed cement [2,3] and the second is to use alkali-activated fly ash (FA), blast furnace slag (BFS), clay, or other aluminosilicate materials [4,5,6,7]. The use of alkali-activated materials can not only save energy but also reduce CO_2_ emissions. At the same time, they can also enhance the durability of concrete and help solve the problem of landfills. This research aims to mix WCP into AAS paste and mortar to study its effect on performance.

Ceramic objects are beautiful and they exhibit an excellent performance, but their waste exerts huge pressure on the environment. Various ceramic objects, such as tiles, roofs, bricks, bowls, and sanitary wares, can be distinguished according to the source of the raw materials used. During ceramic manufacturing and building destruction, a large amount of waste ceramics is generated [8,9]; the proportion of ceramics in construction waste is approximately 45%. The recycling of these wastes is an excellent solution to eliminate waste. In order to achieve the sustainable development goal of low carbon emissions [10], recycling waste ceramics from building materials can help to effectively mitigate their negative environmental effects. This can improve the performance of materials, reduce the waste from industrial plants, reduce energy requirements and production costs, and provide environmental and economic benefits [11,12]. Today, waste ceramics have been used to partially replace natural aggregates in concrete and improve the properties of concrete [13,14], such as its mechanical properties and durability [15,16,17]. However, this limited use of waste ceramics needs to be improved further.

Research on the application of cement with added waste ceramic powder (WCP) has been conducted. The main components of WCP are SiO_2_ and Al_2_O_3_ [11,18]. Li et al. [19] reported that WCP could be used as a pozzolanic supplement for cement. Their results showed that the replacement of cement with WCP can refine the pore structure. In addition, the pozzolanic reaction of WCP produces CASH gel, which can enhance the mutual release of the cement matrix and ceramic particles. Roja et al. [20] also reported similar results and found that WCP can be used as a pozzolanic material. They pointed out that the pozzolanic reaction of WCP arises from the reactive silica and alumina phases. Kannan et al. [18] studied the effect of WCP on the performance of concrete instead of cement. Their results showed that concrete with the addition of WCP had a high strength and excellent durability, and that the WCP was mainly used as a filler rather than as a pozzolanic material.

Although some studies have investigated the application of concrete with waste ceramic aggregate and cement with WCP, a few studies have investigated the use of WCP as the raw material for alkali excitation. Sun et al. [21] reported that WCP can be used as a satisfactory raw material for the creation of geopolymers. They pointed out that the compressive strength of geopolymers at high temperatures was affected by the concentration and content of NaOH, KOH, and Na_2_SiO_3_, and that these exhibited a good thermal stability even when heated up to 1000 °C. Shoei et al. [22] showed that WCP can be used as a raw material for NaOH- and Na_2_SiO_3_-activated geopolymer mortar. They confirmed that the density and compressive strength were affected by the ratio of the alkaline solution to the binder and the curing temperature. In addition, they found that the mortar had the highest strength when the l/b was 0.6 and the curing temperature was 90 °C.

Information on the strength and durability of alkali-activated blast furnace slag (BFS) with added WCP can be obtained from the following studies. Huseien et al. [23,24] studied alkali-activated slag (AAS) with added WCP. The results showed that the formation of the C-(A)-S-H gel was affected by SiO_2_ in the WCP. In addition, adding WCP can improve durability against sulfuric acid attacks. Nailia and Ravil [25] studied AAS with the addition of waste powder made from red clay bricks. They pointed out that the compressive strength was affected by the type of activator, grinding method, and curing conditions used. Keppert et al. [26] reported on a red clay ceramic powder that was prepared using industrial by-products produced during the brick calibration process. They found that when this powder was used as a raw material for geopolymers, the mechanical properties of concrete were affected by the composition of the alkali activator and the CaO content in the powder. A higher CaO content can lead to a wider pore size distribution and a higher porosity. Rashad and Essa [27] reported that adding up to 50% WCP can increase the compressive strength under a curing temperature of 45 °C. At the same time, they reported that the addition of WCP improved the fire resistance of the AAS paste. The change in CaO content (i.e., the Ca/Si ratio) in the geopolymer directly affected the solidification time and compressive strength of the AAS [28]. The Si/Al ratio change affected later strength development [28,29].

Although many studies have been conducted on AAS [4,30], AAS with the addition of WCP has not been extensively studied, and some problems have not been considered in previous studies. Firstly, in previous studies the influence of WCP on the heat of hydration was not considered. Second, in AAS containing WCP, the impact on the durability of chloride ions was not considered. In addition, gypsum formation was not qualitatively analyzed in relation to sulfuric acid corrosion resistance, and the mechanism of sulfuric acid corrosion resistance was not clarified.

Detailed research results on the strength and durability of WCP mixed with AAS paste and mortar are reported in this article. Some experimental studies have been conducted on the fluidity, isothermal calorimetry, compressive strength, ultrasonic pulse velocity (UPV), electrical resistivity, Fourier transform infrared spectroscopy, X-ray diffraction (XRD), resistance to sulfuric acid attack, and non-steady-state chloride ion diffusion coefficient. The corresponding experimental results show the following: (1) As the rate of replacement of WCP increases, the fluidity of the paste increases slightly. (2) As the WCP content increases, the release rate of the early hydration heat will decrease. (3) When WCP is added to mortar, the strength of the mortar sample will decrease in the early period, but no significant decrease in strength will be observed in the later period. (4) As the curing age increases, the value of the UPV will increase. (5) XRD and FTIR spectra show that as the WCP content increases, the gypsum formation will decrease. (6) As the WCP content increases, the durability under sulfuric acid attack will improve. (7) As the WCP content increases, the diffusion coefficient of chloride ions will increase and the resistivity will decrease. From the results of these experimental studies, the relationships between the level of replacement of WCP and the fluidity, heat of hydration, compressive strength, sulfuric acid attack resistance, and chloride ion diffusion coefficient were determined.

## 2. Materials and Test Methods

### 2.1. Raw Materials and Alkali Activators

**Figure 1:**
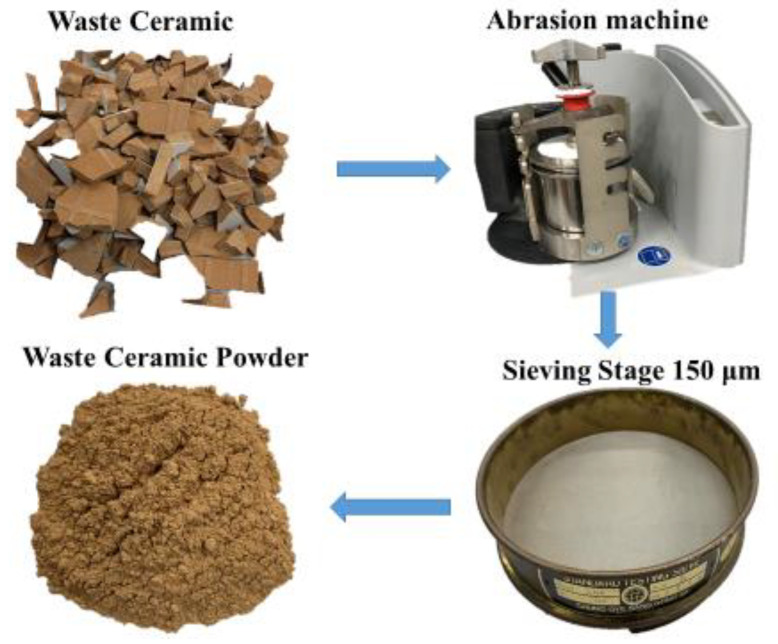
The preparation process of waste ceramic powder (WCP) in the laboratory.

**Figure 2:**
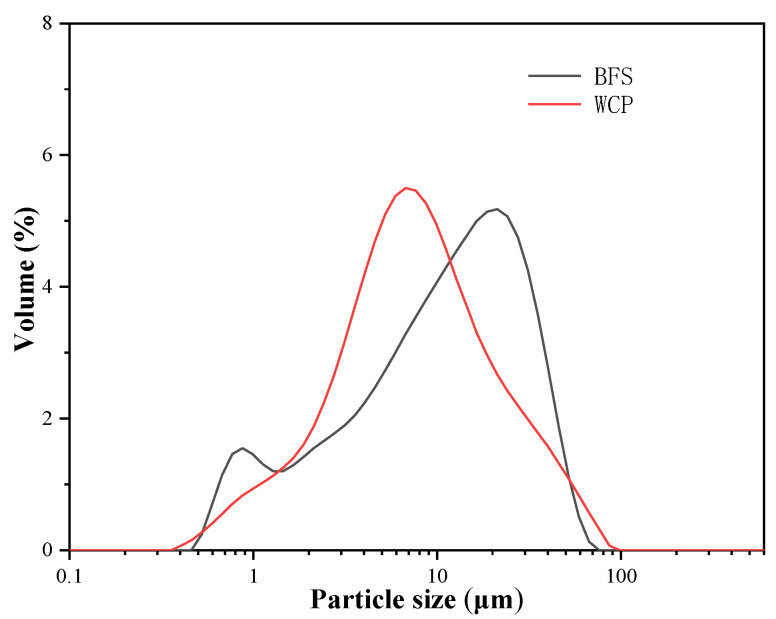
Particle size distribution of blast furnace slag (BFS) and waste ceramic powder (WCP).

In this study, the BFS was supplied by South Korea’s Asia Cement Company (Seoul, Korea). The WCP used in this study was a waste ceramic tile collected during building destruction (Korea). The tile is ground with a ball mill grinder. The process used for obtaining WCP in the laboratory is shown in Figure 1. As shown in Table 1, the main compounds in BFS and WCP are SiO_2_ (32.2% and 66.1%, respectively), CaO (38.9% and 9.32%, respectively), and Al_2_O_3_ (15.7% and 15.9%, respectively). In this study, we found that the CaO percentage of WCP was much lower than that of BFS. The SiO_2_ content was higher than that of BFS. The specific gravities of BFS and WCP measured using the pycnometer method were 2.82 and 2.61, respectively. Through particle size distribution (PSD) analysis (Figure 2), the median particle sizes (D_50_) of BFS and WCP were found to be 12.2 μm and 7.92 μm, respectively. The median particle size of WCP was smaller than that of BFS.

**Figure 3:**
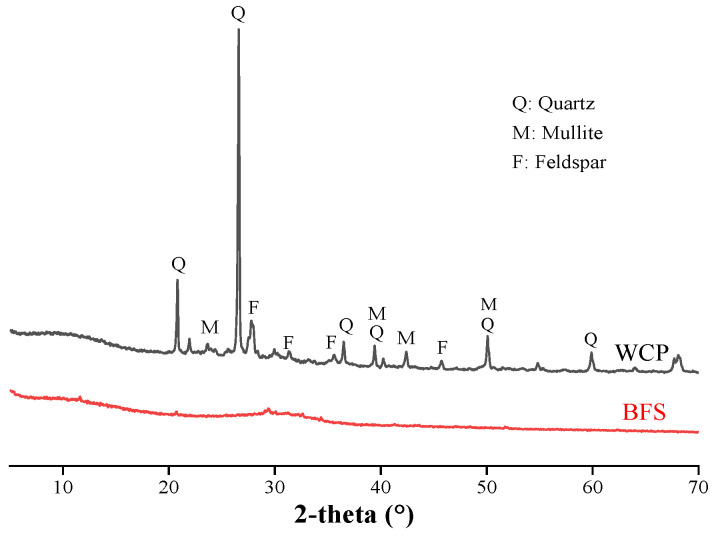
X-ray diffraction (XRD) patterns for blast furnace slag (BFS) and waste ceramic powder (WCP).

The XRD patterns of BFS and WCP are shown in Figure 3. Silica, mullite, and feldspar were the main crystalline components of the WCP. The XRD pattern of BFS did not have any sharp peaks, confirming the extremely amorphous characteristics of the material.

Two types of activators were used to accelerate the reactivity of the mixtures: (1) commercial liquid sodium silicate (SiO_2_ = 29%, Na_2_O = 9.5%, and water = 61.5%) and (2) sodium hydroxide particles with a purity of 98% (GR grade). The sodium hydroxide particles were dissolved in water to produce a 2M sodium hydroxide solution (NH solution). As sodium hydroxide particles generate heat when dissolved in water, they were cooled for 24 h and then mixed with liquid sodium silicate (Na_2_SiO_3_, NS solution) to produce an alkaline solution with a SiO_2_:Na_2_O molar ratio of 1.2 [23].

### 2.2. Mixing Ratio and Sample Preparation

By preparing four types of mixtures, the effect of the use of WCP instead of BFS on the performance of alkali-activated pastes and mortar mixtures was studied. Table 2 lists the detailed mixing information for the PWCP and MWCP samples. Labels beginning with P and M represent paste and mortar samples, respectively. The weight ratios of BFS: WCP used to prepare the PWCP were 100:0, 90:10, 80:20, and 70:30. The names of the above pastes are PWCP0, PWCP10, PWCP20, and PWCP30. MWCP was prepared with a sand/binder ratio of 2. The names of the mortar mixtures are MWCP0, MWCP10, MWCP20, and MWCP30. In this study, ISO standard sand was used as the fine aggregate, the maximum particle size was 2 mm, and the saturated surface dry density was 2.6 g/cm^3^. The PSD of sand met the requirements of ISO 679, and the water absorption rate was less than 0.2%. To ensure the fluidity of the fresh pastes, the ratio of alkali solution/binder (l/b) was 0.55, while the ratio of water/binder (w/b) was 0.46, including water from liquid sodium silicate and additional water. For all the pastes and mortars, the alkali activator accounted for 4% of the adhesive mass based on Na_2_O, which remained constant [31].

In accordance with the ASTM C305 standard, a mechanical stirrer was used to prepare the samples. The uniform pastes and mortars were poured into a cube mold with a size of 50 mm × 50 mm × 50 mm, and the fresh sample was sealed with a film. The sample was then placed in a curing chamber at 20 ± 2 °C. After 48 h of casting, the sample was de-molded, and the sample was wrapped with a film for sealed curing. The curing temperature was 20 ± 2 °C.

### 2.3. Test Method

According to the ASTM-1437 specification, the newly stirred paste must be tested for fluidity. The cone was placed at the center of the steel plate and the paste was slowly poured into it. The cone was vibrated 20 times and the top surface of the mold was polished smooth with a scraper. The vertebral body was then lifted perpendicular to the plate. The diameters of the two vertical intersections were measured and averaged.

Using the TAM Air isothermal calorimeter (TA Instruments Delaware, New Castle, DL, USA), an isothermal calorimetry experiment was conducted at 20 °C to measure the heat of hydration in the first 168 h. To minimize the impact of environmental heat on the sample, the indoor temperature was maintained at 20 °C [32]. Using a medicine spoon, 3.226 g of BFS and WCP powders were weighed, mixed evenly, and placed into an ampoule; then, a plastic dropper was used to drop and thoroughly mix 1.774 g of mixed alkali solution. Finally, the ampoule was quickly placed in the calorimeter.

For the UPV and compressive strength, a sample with a size of 50 mm × 50 mm × 50 mm was used. In accordance with the ASTM C519 specification, a portable ultrasonic tester (Pundit Lab, Proceq, Switzerland) was used for the non-destructive testing of mortar samples cured for 3, 7, and 28 days. Three samples of each mortar were used to determine the average value. After the UPV test, the compressive strength of the mortar sample was tested in accordance with the ASTM C349 specifications. Three samples of each mortar were tested to determine their average values.

The Proceq Resipod resistivity tester produced in Switzerland was used to perform non-destructive testing on cylinders with a diameter of 100 mm and a height of 200 mm on 3-, 7-, and 28-day mortar samples. The test was conducted in strict accordance with the AASTO T 358 specifications.

A Frontier spectrometer (PerkinElmer, Waltham, MA, USA) was used to measure the Fourier transform infrared spectroscopy (FTIR) spectra of the samples (cured for 3 days and 28 days) with a resolution of 0.4 cm^−1^. The wavenumber was in the range 4000–500 cm^−1^ at room temperature, and each sample was scanned 32 times.

XRD analysis was performed on the samples. PANalytical X’pert pro-MPD diffractometers with Cu Kα radiation (λ = 1.5407 Å) were used. The working voltage and current used were 40 kV and 30 mA, respectively. Scans were performed from 5° to 70° with a step length of 0.013°, and the cumulative scan time for each step was 8.67 s.

In accordance with ASTM C267, a 10% H_2_SO_4_ solution for industrial use was selected to attack the mortar samples [23]. After 28 days of curing, 12 samples were selected and weighed for each mortar sample. Six samples were then immersed in H_2_SO_4_ solution and the other six samples were placed in deionized water as a control group. To keep the pH value of the acidic solution stable, the H_2_SO_4_ solution was replaced every two weeks throughout the entire experiment. All the mortar samples were tested after 28 and 180 days, including XRD and FTIR testing. At the same time, in accordance with the ASTM C267 specification [33], two different factors were considered to evaluate the performance: the weight change and residual strength.

The diffusion coefficient of the chloride ions was measured in accordance with the ASTM C1202-97 specifications. A cylindrical mortar sample with a diameter of 100 mm and a length of 200 mm was poured, using the mixing ratio shown in Table 2. At 28 days of age, a 50 mm-thick section was cut from the center of the cylinder. The two diffusion cells (anode and cathode) were filled with 0.3 N NaOH and 3% NaCl solutions, and a voltage of 30 ± 0.2 V was applied [34]. The duration was 24 h. After the test, the test piece was split from the middle and sprayed with 0.1 N AgNO_3_ solution, then the depth of the discolored part was measured. The diffusion coefficient of mortar was calculated using Equation (1) [35]:

(1) $$D_{nssm\;}=\frac{RTL}{zFU}\frac{x_{d}-\alpha \sqrt{x_{d}}}{t_{d}},$$

where $D_{nssm\;}$ is the unsteady migration diffusion coefficient (m^2^/s), $\;x_{d}$ is the diffusion depth of chloride ions (m), is the test duration (s), and the test constant α is calculated using Equation (2) [34]:

(2) $$\alpha =2\sqrt{\frac{RTL}{zFU}}erf^{-1}\left(1-\frac{2c_{d}}{c_{0}}\right),$$

where  R is the gas constant R  = 8.314 J/K mol; T is the test temperature of the cathode (K); L is the sample thickness (m); z is the absolute value of the valence of the chloride ion; F is the Faraday constant = 96.5 KJ/V mol; U is the absolute value of the applied voltage; $c_{0}$ is the concentration of the catholyte chloride ion; and cd is the concentration of chloride when the color of the silver nitrate solution changes: $c_{d}=0.07N$.

For the above tests, suitable paste and mortar samples were selected for testing. The test items and test times are listed in Table 3.

## 3. Results and Discussion

### 3.1. Flow Characteristics

**Figure 4:**
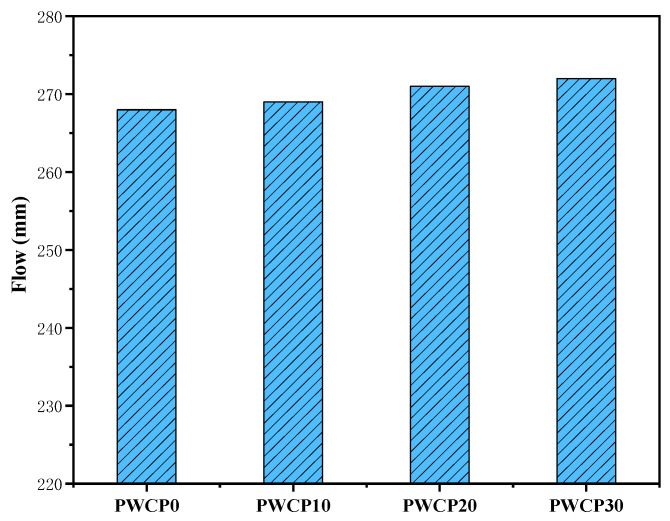
Effect of the waste ceramic powder (WCP) content on the flow value of the mixtures.

Many factors affect the flow characteristics of AAS, such as the liquid/slag ratio, the type of activator used, the water glass modulus, and additives. This study considered only the impact of WCP substitution on the flow. The flow values of the freshly prepared pastes are shown in Figure 4. As the WCP content increased, the flow value of the freshly prepared pastes increased slightly. Compared with the control group WCP0, the flow value of WCP10, WCP20, and WCP30 increased by 0.37%, 1.12%, and 1.49%, respectively. The increased flow value of the pure paste may be due to the different physical properties and chemical reactions of the raw materials [36]. Compared with WCP, BFS can provide more reactive materials, with the result that rapid agglomeration leads to a low flow value of PWCP0 [23].

### 3.2. Hydration Heat

**Figure 5:**
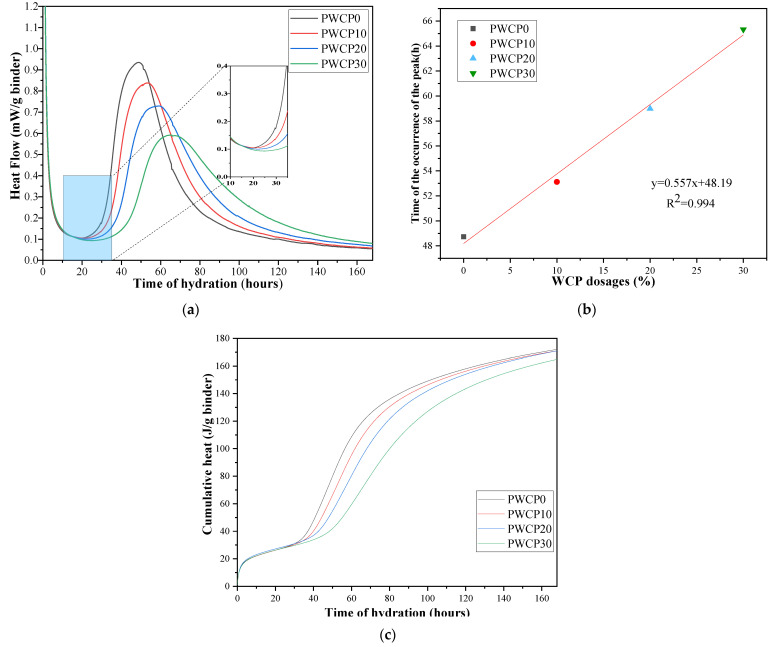
(**a**) Normalized heat flows of PWCP. (**b**) Effect of different waste ceramic powder (WCP) dosages on the time of the occurrence of the peak. (**c**) Cumulative hydration heat of PWCP.

Figure 5a shows the curve of the release rate of the heat of hydration within 168 h of the AAS measurement. In previous studies, the hydration heat release curves of cement-based materials and alkali-excited materials have been divided into five stages: the initial hydration period, induction period, acceleration period, deceleration period, and stable reaction period [37]. The hydration heat release rate curve shows that, when the binder is mixed with the alkali solution, heat is released. This is caused by the reaction between the silicate provided by the alkaline solution and the calcium ions dissolved in the BFS, which produces the CSH phase [38]. Most of the dissolution of BFS occurred during this period, and the heat of dissolution was the main reason for the first peak of the hydration heat [37]. During the initial hydration and induction periods, the hydration exothermic curves of all the samples were almost the same, which indicates that the dissolution of BFS was not affected by the WCP content. As shown in Figure 5a, the amount of WCP present after the induction period is inversely proportional to the rate of heat release, which indicates that the inclusion of WCP in the binder affects the reaction kinetics at the initial stage of curing at 20 °C under the alkali excitation conditions used. This may be because the presence of WCP reduces the agglomeration of BFS. This shows that the curing conditions at 20 °C are not sufficient to promote the extensive reaction of WCP in the early stage.

The replacement rates of WCP were 10%, 20%, and 30%, respectively. Figure 5a shows that as the WCP replacement rate increases, the peak hydration heat release rate exhibits the same trend and delay. Figure 5b shows a good linear relationship between the time when the hydration exothermic rate peak appears and the WCP content, with an R^2^ value of 0.994.

Figure 5c shows the cumulative heat of hydration within 168 h of AAS. The accumulated heats of hydration at 72 h for WCP0, WCP10, WCP20, and WCP30 were 128.2, 120.34, 108.38, and 84.92 J/g, respectively. The cumulative heat of all the samples was almost the same at 168 h, which indicates that the chemical exothermic reaction occurred when WCP was added to the AAS. The active silica in the WCP may have reacted with the alkali to release heat.

### 3.3. Compressive Strength

It is well known that many factors affect the compressive strength of AASs, such as the curing temperature, the alkali concentration, and the SiO_2_:Na_2_O molar ratio [39]. This section evaluates and discusses the effect of adding WCP on the compressive strength of alkali-activated mortar. The compressive strength of the mortar when the replacement rate of the WCP mass was 10%, 20%, and 30% was evaluated and compared with a control group containing 100% BFS.

**Figure 6:**
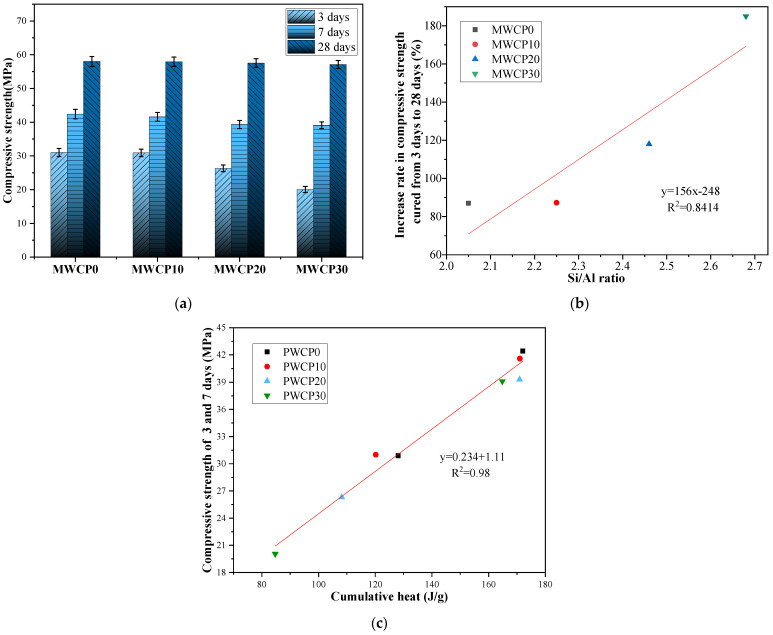
(**a**) Effect of the replacement of blast furnace slag (BFS) with waste ceramic powder (WCP) on the compressive strength. (**b**) The relationship between the rate of increase in the compressive strength and the Si/Al ratio. (**c**) Relationship between compressive strength and cumulative heat at 3 and 7 days.

Figure 6a summarizes the compressive strength test results of the mortar samples. It can be seen from the results that the early strength of the mortar with added WCP is lower than that of the control group. At 3 days, the control group (MWCP0) provided the highest compressive strength (31 MPa), and as the WCP replacement rate increased the compressive strength decreased. This decrease in the early compressive strength may be related to the lower CaO content in the WCP [23], which changes the Ca/Si ratio and negatively affects the compressive strength. In addition, the low CaO content in WCP also affects the production of CASH gel, which can slow down the early chemical reaction rate, as confirmed by the hydration heat results (Section 3.2). In summary, the main factor affecting the early compressive strength is the difference in the chemical composition of the raw materials (BFS and WCP), which greatly affects the alkali-activated process. As alkali activation progresses, the compressive strength increases monotonically with the increase in curing age. The rate of increase β of the compressive strength is calculated by Equation (3) [40]:

(3) $$\beta =\left(\frac{P_{28}}{P_{3}}-1\;\right)\times 100\%,$$

where $P_{3}$ and $P_{28}\;$ are the compressive strength values of the mortar cured for 3 and 28 days, respectively.

The rates of increase in the compressive strength at 28 days (MWCP0, MWCP10, MWCP20, and MWCP30) compared with the strength curing for 3 days were calculated as 87%, 87.3%, 118%, and 185%, respectively. This may be because the reactive silica in the later WCP participated in the reaction. The introduction of WCP changed the initial Si/Al ratio in the mixture and determined the relative amounts of AlO_4_ and SiO_4_ in the CASH gel. The initial Si/Al ratio (γ) in the mixture can be calculated using Equation (4) [41]:

(4) $$\Gamma =\left[\frac{a\times \left(1-w\right)+bw}{c\times \left(1-w\right)+dw}\right]$$

where a  and b are the mass percentages of SiO_2_ in BFS and WCP, respectively; c and d are the mass percentages of Al_2_O_3_ in BFS and WCP, respectively; and w is the replacement rate of WCP in the mixture.

The Si/Al ratios of MWCP0, MWCP10, MWCP20, and MWCP30 were calculated as 2.05, 2.25, 2.46, and 2.68, respectively. Figure 6b shows the relationship between the rate of increase in the compressive strength of the mortar and the Si/Al ratio. It can be seen from the results that, compared with the mortar cured from 3 days to 28 days, the rate of increase in the compressive strength increases as the initial Si/Al ratio in the mixture increases. There was an obvious linear relationship between the rate of increase in the compressive strength of the mortar and the initial Si/Al ratio—that is, the value of R^2^ was 0.84. This is consistent with the results reported in the literature [28]. In the mixture, increasing the percentage of initial active silica is beneficial for the formation of CASH gels with a high Si/Al ratio [29,42]. The presence of active silica enhances the geopolymer process and introduces more silicon into the polymer chain, which enhances the later strength.

WCP has positive and negative effects on the compressive strength of AAS mortar. The positive effect is that, compared with mortar cured from 3 days to 28 days, the WCP-containing mortar increases the rate of increase in the compressive strength, which is attributed to the participation of the active silica in the reaction in the WCP. The negative effect of this is that the early strength decreases with the increase in WCP content, which is attributed to the lower CaO content of WCP.

Figure 6c shows the relationship between the cumulative heat and the compressive strength at 3 and 7 days. It was found that there was an obvious linear relationship between the cumulative heat and the compressive strength at 3 and 7 days, and the value of R^2^ was 0.98. As we know, the data obtained by experiments will always have certain differences. Even if repeated experiments or sampling are performed under the same conditions, the final data values obtained will be different. The level of variance of a particular test can be determined based on the results of individual tests, average test results, and the number of tests performed. Moreover, the coefficient of variance can be determined using variance and the average value can be obtained. In this study, error bars were added to show the experimental data when processing the experimental data. The coefficient of variance of the results of the strength tests was less than 5%.

### 3.4. UPV

The UPV test is a non-destructive test method that is used to determine and evaluate the characteristics of concrete [7]. This test method is based on measuring the propagation time of an ultrasonic pulse through the concrete to be tested. The UPV can be obtained by dividing the propagation path length of the ultrasonic pulse by the propagation time.

**Figure 7:**
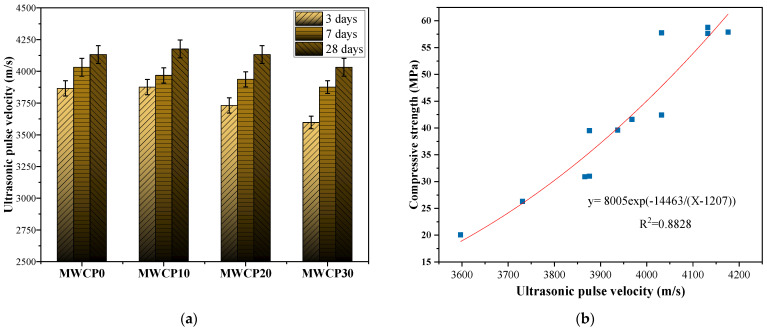
(**a**) Ultrasonic pulse velocity (UPV) varies with curing age. (**b**) Relationship between UPV and compressive strength.

For cement concrete materials, the UPV test is affected by w/b, age, cement content, water, and aggregate properties; therefore, the pulse speed and propagation path vary [43]. Figure 7a shows the UPV test results of the mortar samples at ages of 3, 7, and 28 days. As the maintenance age increases, the value of the UPV also increases. As the curing age increases, the alkaline solution is consumed, and the sizes of the pores in the sample decrease [43]. This trend is similar to the results of the compressive strength test, in that the values of UPV and compressive strength both increase with age. Figure 7b shows the relationship between the UPV and the compressive strength. It can be seen that they have an exponential relationship, and the R^2^ value is 0.8828. In the literature [43], it can be seen that there is an exponential relationship between UPV and compressive strength, with R^2^ being measured to be between 0.927 and 0.991, which is similar to our results. The coefficient of variance of the test results of UPV is less than 2%.

### 3.5. FTIR

**Figure 8:**
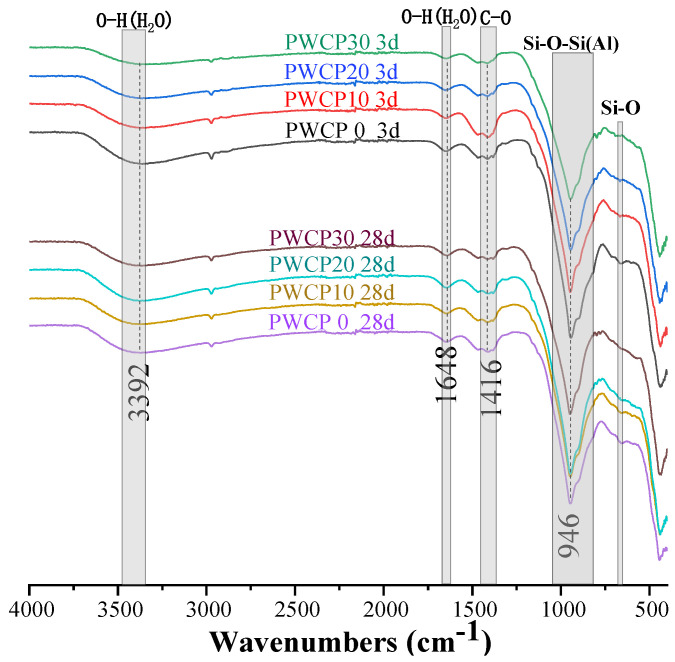
Fourier transform infrared spectroscopy (FTIR) spectra of PWCP sample at 3 and 28 days.

Figure 8 shows the FTIR spectra of all PWCP samples at 3 and 28 days. Table 4 lists the wavenumber characteristics of the related functional groups. The absorption peak with a wavenumber of 3392 cm^−1^ is the stretching vibration of the O–H chemical bond [44,45]. The bending vibration of the O–H bond appears in the range of 1650–1640 cm^−1^ [44,45]. The peaks in the wavenumber ranges of 997–938 cm^−1^ and 853–600 cm^−1^ correspond to the Si–O–Si (Al) asymmetric stretching vibration and the Al–O–H bending vibration [46,47]. The peak at 1465 cm^−1^ is related to the asymmetric stretching vibration of the C–O bond. The absorption peak of the stretching vibration of the Si–O bond in SiO_2_ was in the range of 670–661 cm^−1^ [48]. The presence of the O–H bond absorption peak may be related to the alkali solution in the mixture. Moreover, the pronounced intensity of the Si–O bond absorption peak may be attributed to the fact that the increase in the amount of substitution of WCP increases the SiO_2_ content. The presence of the C–O bond absorption peak may be explained by the fact that the sample was carbonized during the preparation process.

After curing for 3 days, the WCP content increased, weakening the absorption peak intensity of the Si–O–Si (Al) bond in the paste. This shows that the CASH gel formation was reduced. However, no obvious change in the absorption peak of the Si–O–Si (Al) bond occurred in the paste after 28 days. This means that the content of CASH was approximately the same in each sample.

### 3.6. XRD Analysis

**Figure 9:**
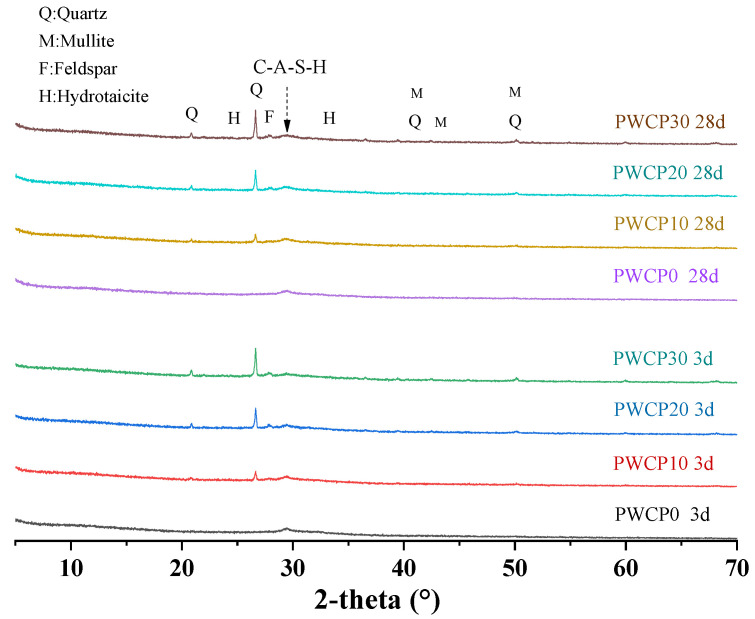
Effect of waste ceramic powder (WCP) replacing blast furnace slag (BFS) on the X-ray diffraction (XRD) results of PWCP.

Figure 9 shows the XRD results for different WCP compositions after 3 and 28 days. It should be noted that the silica in the WCP is composed of an amorphous phase and a crystalline phase, and only the amorphous-phase active silica participates in the polymerization reaction. The peak of the hydrotalcite (Mg_6_AlCO_3_OH_16_·4H_2_O) phase may be difficult to identify because of its small number and low crystallinity [50]. As the WCP content increased, the peak intensity of the hydrotalcite phase decreased. This is because of the shallow penetration of Mg into the WCP, which led to a reduction in the hydrotalcite phase. For the PWCP sample containing WCP, quartz, mullite, and feldspar were detected, and the peak intensity increased with the increase in the WCP content, which was always present owing to the inertness of the quartz reaction. In addition, a hump of CASH was also observed. Since CASH is an amorphous phase, it is difficult to observe changes in the amount of CASH across different samples.

### 3.7. Sulfuric Acid Attack

**Figure 10:**
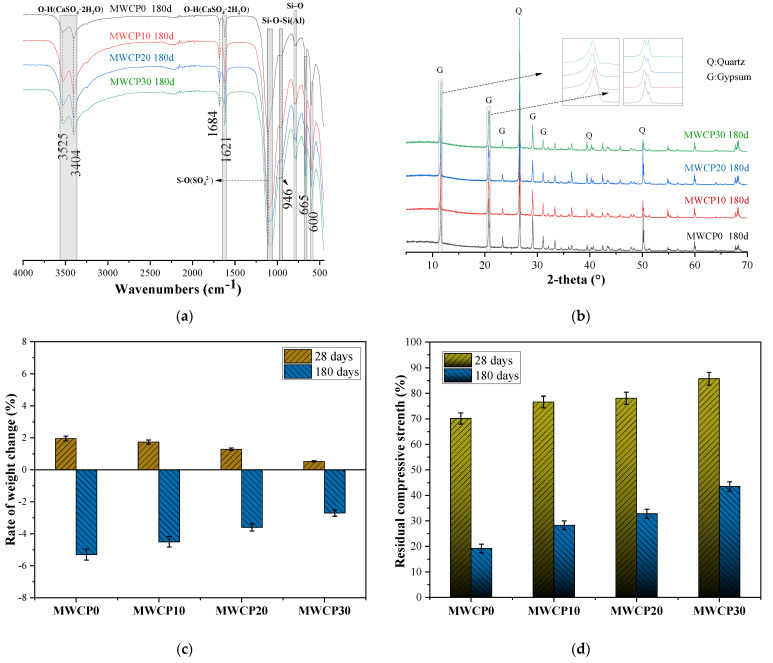
(**a**) Fourier transform infrared spectroscopy (FTIR) spectra of MWCP sample after 180 days of 10% sulfuric acid attack. (**b**) X-ray diffraction (XRD) results of MWCP exposed to 10% sulfuric acid for 180 days. (**c**) The weight change rate of prepared MWCP exposed to 10% H_2_SO_4_. (**d**) Percentage of residual strength of prepared MWCP exposed to 10% H_2_SO_4_.

Figure 10a shows the FTIR spectrum of the MWCP samples exposed to 10% sulfuric acid. The absorption peak position clearly proves the presence of gypsum in the MWCP sample attacked by sulfuric acid. The peaks appearing in the range of 3550–3400 cm^−1^ with wavenumbers of 1684 cm^−1^ and 1621 cm^−1^ are attributed to the stretching and bending vibrations of the O-H bond of gypsum crystals, respectively [49]. The tensile vibration peaks of the S–O (SO_4_^2−^) bond appeared in the wavenumber range of 1120–1133 cm^−1^, while the bending vibration of the S–O bond appeared near the wavenumbers 1684 cm^−1^ and 1621 cm^−1^ [49]. Compared with the FTIR spectrum of the PWCP sample that was cured for 28 days, the absorption peak intensity of the Si–O–Si (Al) bond became weaker after the mortar was attacked by sulfuric acid for 180 days. This showed that the Si–O–Si (Al) chemical bond is vulnerable to acid attack and rupture. At the same time, it also shows that the CASH gel decomposed.

Figure 10b shows the XRD results of the mortar exposed to 10% sulfuric acid for 180 days. After the mortar was eroded by sulfuric acid for 180 days, XRD detected a large amount of gypsum phase (CaSO_4_·2H_2_O). As the WCP content increased, the intensity of the gypsum peak decreased. When calcium-rich substances are exposed to sulfuric acid, gypsum is formed [49]. This is because the sulfate anion in the solution infiltrates the sample and reacts with the dissolved calcium cation. As the BFS is insoluble in acid solution [51], the Ca^2+^ in the pore solution may originate from the decalcification reaction of the CASH gel.

Previous experimental results have shown that the WCP content could strongly affect the freshness and hardening properties of PWCP and MWCP over time. Sulfuric acid attacks mortar samples mainly by dissolving the binder, which weakens the strength of the affected mortar [23]. The change in the mass of the mortar sample after exposure to a 10% H_2_SO_4_ solution for 28 days and 180 days is shown in Figure 10c. After immersion in acid solution for 28 days, the mass of all the mortar samples increased. However, after immersion in sulfuric acid solution for 180 days, the mass of all the mortar samples decreased. The mass change results show that in the early stage of immersion in the sulfuric acid solution, the dissolution caused by H^+^ lagged behind that caused by sulfate [52]. H^+^ mainly causes dissolution and mass loss, whereas the gypsum formed by the reaction of sulfate and calcium ions causes a mass increase and eventually mass loss due to excessive expansion, cracking, and peeling. The combined effect of dissolution and swelling led to the deterioration of the mortar, which led to a loss of mortar mass.

Figure 10d shows the percentage of residual strength after immersion in a 10% sulfuric acid solution for 28 and 180 days. As the WCP content increased, the strength loss gradually decreased. When the mortar samples were soaked in the sulfuric acid solution for 28 days, the residual strengths were 70.14%, 76.59%, 78.06%, and 85.73%, respectively. When the immersion time was extended to 180 days, the strength loss accelerated, and the residual strengths became 19.2%, 28.3%, 32.8%, and 43.5%. The decrease in strength can be summarized as follows: First, the C-A-S-H gel decalcifies and decomposes, contributing significantly to the strength loss. Second, the gypsum causes the expansion of the alkali-activated mortar, generating additional cracks in the sample that cause further deterioration.

After immersion in the sulfuric acid solution for 180 days, compared with all mortar samples, the control group (MWCP0) had the largest mass loss and the lowest residual strength, while MWCP30 had the smallest mass loss and the largest residual strength. Compared with the mortar samples containing WCP, the high CaO content in the control (100% BFS) samples resulted in greater gypsum formation [23]. The high gypsum content increased the expansion of the sample, increased the sizes of the cracks, increased the spalling, increased the mass loss, and reduced the compressive strength. Therefore, as the WCP content increased, the corrosion resistance to sulfuric acid increased. In addition, when testing the weight change and residual strength, there are also differences between different specimens due to the unevenness of the specimens. When processing experimental data, error bars are added. The coefficient of variance of the test results of the weight change is less than 8%. The coefficient of variance of the tests results for residual strength is less than 9%.

### 3.8. Resistivity

**Figure 11:**
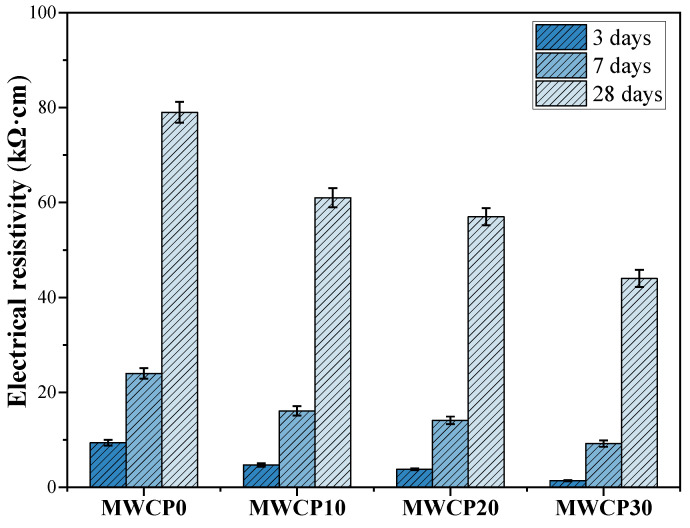
Electrical resistivity of MWCP at curing ages of 3, 7, and 28 days.

Resistivity is an important parameter for the evaluation of concrete structures. Resistivity can serve as a good measure of the interconnectivity of voids in a matrix and the resistance of concrete to chemical attack and steel corrosion [53]. Figure 11 shows the resistivity results of all MWCP samples tested by the four-electrode Wenner method after curing for 3, 7, and 28 days. This study found that with an increase in age, the resistivity of all the samples increased. As the reaction progressed, the ions in the alkaline solution were consumed. CASH bound more alkali ions, thereby increasing the resistivity [54]. At the same time, a pH drop also increased the resistivity [55]. For samples of the same age, the resistivity decreased with an increase in WCP content. Moreover, the increase in WCP content could affect unreacted and partially reacted particles, which can produce more porous structures [23]. These porous structures led to an increase in porosity and a decrease in resistivity. When testing resistivity, the values displayed for different specimens of the same samples are also different. Therefore, when processing experimental data, error bars are added. The coefficient of variance for the test results of resistivity is less than 7%.

### 3.9. Non-Steady-State Diffusion Coefficients of Chloride Ions

**Figure 12:**
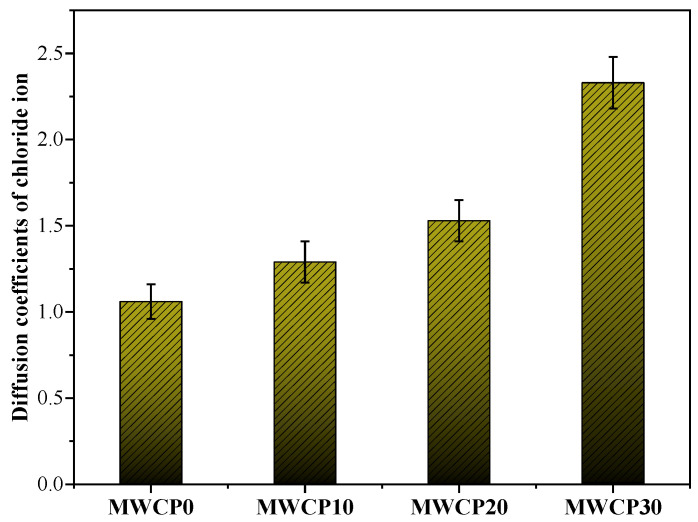
Effect of waste ceramic powder (WCP) content on the diffusion coefficients of chloride ions.

Chloride ions are one of the main reasons for the corrosion of steel bars, and they decrease the durability of concrete structures. The permeability of chloride ions is an important parameter for evaluating the resistance of concrete to chloride corrosion. The corrosion of steel bars is related to concrete cracking and spalling, especially for buildings exposed to marine environments and roads where deicing salt is spread [56]. A voltage was applied to accelerate the diffusion of chloride ions, and AgNO_3_ was used to indicate the depth of chloride ion diffusion. However, for the control group (100% BFS), it is difficult to observe the formation of silver chloride because of the shallow penetration of chloride ions into the AAS. The penetration depth increased with the increase in the WCP content in MWCP. Figure 12 shows the non-steady-state diffusion coefficients of the chloride ions of MWCP calculated from the experimental data. When we tested the depth of penetration of chloride ions in this study, the depth of penetration in different parts of the same sample was also different. Therefore, when processing the experimental data, error bars were added to display the calculated chloride ion diffusion coefficient. The coefficient of variance for the test results for the diffusion coefficient is less than 10%. The largest chloride ion diffusion coefficient was observed in the sample MWCP30. The increase in the chloride diffusion coefficient can be attributed to an increase in the porosity. An increase in the WCP content will affect unreacted and partially reacted particles, resulting in more porous structures [23]. This increases the porosity and diffusion coefficient and decreases the chloride ion durability.

### 3.10. Relationship between Sulfuric Acid Durability and Chloride Ion Durability

The analysis of the results of sulfuric acid attack shows that as the WCP content increased, the gypsum formation decreased, and the sulfuric acid durability increased. The chloride ion diffusion coefficient results show that as the WCP content increased, the diffusion depth of the chloride ions increased and the chloride ion durability decreased. That is, sulfuric acid’s durability and the tendency of chloride ions to diffuse opposed each other. In simple terms, from the porosity and the diffusion coefficient results, we can state that as the chloride ion diffusion coefficient increased, the sulfate ion diffusion coefficient increased. This paradoxical result is related to the formation of gypsum, which causes mortar to swell. Gypsum formation depends on two factors: sulfate ions and calcium ions. As the diffusion coefficient increases, the supply of sulfate ions increases. However, as the WCP content increases, the supply of calcium ions decreases and the reaction between sulfate ions and calcium ions is restricted, which ultimately decreases the gypsum formation, weakening the deterioration caused by swelling and improving sulfuric acid’s corrosion resistance.

### 3.11. Relationship between Compressive Strength, Resistivity, and Chloride Ion Durability

The analysis of the compressive strength showed that after 28 days of curing, no significant decrease in the compressive strength was observed with the increase in WCP content. After 28 days of curing, the results of the electrical resistivity and chloride ion durability analyses showed that with the increase in WCP content, the electrical resistivity decreased, and the chloride ion diffusion coefficient increased. The decrease in electrical resistivity and the increase in chloride ion diffusion coefficient may have been caused by the increase in porosity as the WCP content increased. In other words, after curing for 28 days, paradoxical results were obtained for the compressive strength, resistivity, and chloride ion durability. The explanation for this is that the influence of the Si/Al ratio in the mixture on the compressive strength plays the primary role, while other factors play a secondary role.

## 4. Conclusions

WCP, unlike other geopolymers, is obtained by recycling building materials. The experiments conducted in this study yielded the following conclusions:

1. As the WCP content increased, the flow value of the paste increased slightly, which may be due to the varying physical properties and chemical reactions of the raw materials.

2. The results of the heat of hydration analysis show that as the WCP content increased, the cumulative heat of hydration over 72 h decreased. This is because the WCP reaction rate is slower in the early stages. The cumulative heat of hydration for 168 h was almost the same, which shows that WCP participated in the reaction.

3. The UPV and strength analysis results show that when WCP was added to the mortar, the UPV and strength of the mortar sample decreased in the early stage, but no significant decrease in UPV and strength was observed in the late curing stage. The reason for the decrease in UPV and strength in the early stage may be related to the lower CaO content in WCP, while the UPV and compressive strength did not change significantly in the later stage. It may be that the active silica in the WCP participated in the reaction.

4. The FTIR and XRD analysis results show that as the WCP content increased, the peak intensity of the hydrotalcite phase decreased. This may be due to the lower Mg content of the WCP.

5. The use of WCP as a substitute for BFS in an alkali-activated matrix can improve the durability of mortar exposed to sulfuric acid attack. As the WCP content increased, the strength loss gradually decreased. This is because as the supply of the calcium source is reduced, gypsum formation is reduced, and the deterioration caused by gypsum is reduced.

6. The use of waste ceramic powder as a substitute for BFS in an alkali-activated matrix improved the diffusion coefficient of chloride ions and reduced the resistivity. This may be related to the increase in the WCP content, resulting in the development of more porous structures.

## Figures and Tables

**Table 1:** Chemical and physical properties of blast furnace slag (BFS) and waste ceramic powder (WCP).

Material	Chemical Compositions (wt%)										Density (g/cm^3^)
	CaO	SiO_2_	Al_2_O_3_	Fe_2_O_3_	MgO	SO_3_	K_2_O	Na_2_O	ZnO	LOI ^1^	
BFS	38.9	32.2	15.7	0.65	7.08	2.65	0.61	0.30	—	1.25	2.82
WCP	9.32	66.1	15.9	2.38	0.58	0.42	1.93	1.12	0.15	1.19	2.61

^1^ Loss on ignition.

**Table 2:** Paste and mortar mixture samples.

Specimens	Binder		Alkali Activator		Sand	Water	w/b	l/b
	BFS	WCP	NaOH	Water Glass				
PWCP0	100	0	3.2	16	0	35.8	0.46	0.55
PWCP10	90	10						
PWCP20	80	20						
PWCP30	70	30						
MWCP0	100	0	3.2	16	200	35.8	0.46	0.55
MWCP10	90	10						
MWCP20	80	20						
MWCP30	70	30						

**Table 3:** Experimental methods and scope.

Method	Test Samples	Test Time
Fluidity	Paste	-
Compressive strength	Mortar	3, 7, and 28 days
Ultrasonic pulse velocity	Mortar	3, 7, and 28 days
Resistivity	Mortar	3, 7, and 28 days
Hydration heat	Paste	168 h
Fourier transform infrared spectroscopy spectra	Paste	3 and 28 days
X-ray diffraction	Paste	3 and 28 days
Sulfuric acid attack	Mortar	28–208 days
Chloride ion penetrability	Mortar	28 days

**Table 4:** Positions of infrared bands and functional groups in attenuated total reflection-Fourier transform infrared spectroscopy (ATR-FTIR) spectra.

Wavenumber (cm^−1^)	Functional Groups	References
3550–3400	H–O–H (CaSO_4_·2H_2_O), υ	[49]
3457–3390	H–O–H (H_2_O), υ	[44,45]
1650–1640	H–O–H (H_2_O), δ	[44,45]
1133–1120	S–O (SO_4_^2−^), υ	[49]
997–938	Si–O–Si (Al), υ_as_	[46,47]
853–600	Al–O–H, δ	[46]
670–661	Si–O, υ	[48]

## Data Availability

The data presented in this study are available on request from the corresponding author.
